# Continuous-capture microwave imaging

**DOI:** 10.1038/s41467-021-24219-0

**Published:** 2021-06-25

**Authors:** Fabio C. S. da Silva, Anthony B. Kos, Grace E. Antonucci, Jason B. Coder, Craig W. Nelson, Archita Hati

**Affiliations:** 1 National Institute of Standards and Technology, Boulder, CO USA; 2 Wavsens, Westminser, CO USA; 3 University of Colorado, Boulder, CO USA

**Keywords:** Applied mathematics, Microwave photonics, Imaging and sensing, Imaging techniques

## Abstract

Light-in-flight sensing has emerged as a promising technique in image reconstruction applications at various wavelengths. We report a microwave imaging system that uses an array of transmitters and a single receiver operating in continuous transmit-receive mode. Captures take a few microseconds and the corresponding images cover a spatial range of tens of square meters with spatial resolution of 0.1 meter. The images are the result of a dot product between a reconstruction matrix and the captured signal with no prior knowledge of the scene. The reconstruction matrix uses an engineered electromagnetic field mask to create unique random time patterns at every point in the scene and correlates it with the captured signal to determine the corresponding voxel value. We report the operation of the system through simulations and experiment in a laboratory scene. We demonstrate through-wall real-time imaging, tracking, and observe second-order images from specular reflections.

## Abstract (web summary)

The authors present a microwave imaging system that can operate in continuous transmit-receive mode. Using an array of transmitters, a single receiver and a reconstruction matrix that correlate random time patterns with the captured signal, they demonstrate real-time imaging and tracking through a wall.

## Introduction

Advanced uses of time in image rendering and reconstruction have been the focus of much scientific research in recent years. The motivation comes from the equivalence between space and time given by the finite speed of light *c*. This equivalence leads to correlations between the time evolution of electromagnetic fields at different points in space. Applications exploiting such correlations, known as time-of-flight (ToF)^1^ and light-in-flight (LiF)^2^ cameras, operate at various regimes from radio^3,4^ to optical^5^ frequencies. Time-of-flight imaging focuses on reconstructing a scene by measuring delayed stimulus responses via continuous wave, impulses or pseudo-random binary sequence (PRBS) codes^1^. Light-in-flight imaging, also known as transient imaging^6^, explores light transport and detection^2,7^. The combination of ToF and LiF has recently yielded higher accuracy and detail to the reconstruction process, especially in non-line-of-sight images with the inclusion of higher-order scattering and physical processes such as Rayleigh–Sommerfeld diffraction^8^ in the modeling. However, these methods require experimental characterization of the scene followed by large computational overheads that produce images at low frame rates in the optical regime. In the radio-frequency (RF) regime, 3D images at frame rates of 30 Hz have been produced with an array of 256 wide-band transceivers^3^. Microwave imaging has the additional capability of sensing through optically opaque media such as walls. Nonetheless, synthetic aperture radar reconstruction algorithms such as the one proposed in ref. ^3^ required each transceiver in the array to operate individually thus leaving room for improvements in image frame rates from continuous transmit-receive captures. Constructions using beamforming have similar challenges^9^ where a narrow focused beam scans a scene using an array of antennas and frequency modulated continuous wave (FMCW) techniques.

In this article, we develop an inverse light transport model^10^ for microwave signals. The model uses a spatiotemporal mask generated by multiple sources, each emitting different PRBS codes, and a single detector, all operating in continuous synchronous transmit-receive mode. This model allows image reconstructions with capture times of the order of microseconds and no prior scene knowledge. For first-order reflections, the algorithm reduces to a single dot product between the reconstruction matrix and captured signal, and can be executed in a few milliseconds. We demonstrate this algorithm through simulations and measurements performed using realistic scenes in a laboratory setting. We then use the second-order terms of the light transport model to reconstruct scene details not captured by the first-order terms.

We start by estimating the information capacity of the scene and develop the light transport equation for the transient imaging model with arguments borrowed from basic information and electromagnetic field theory. Next, we describe the image reconstruction algorithm as a series of approximations corresponding to multiple scatterings of the spatiotemporal illumination matrix. Specifically, we show that in the first-order approximation, the value of each pixel is the dot product between the captured time series and a unique time signature generated by the spatiotemporal electromagnetic field mask. Next, we show how the second-order approximation generates hidden features not accessible in the first-order image. Finally, we apply the reconstruction algorithm to simulated and experimental data and discuss the performance, strengths, and limitations of this technique.

## Results

### Imaging model

**Fig. 1:**
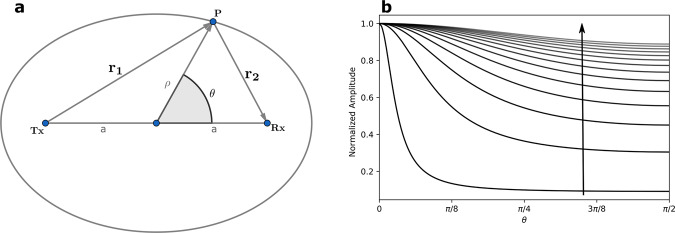
Position evaluation based on time of flight. In panel **a**, a diagram showing an ellipse with focal points **Tx** and **Rx** as the solution to the equation *r*_1_ + *r*_2_ = *r* and the corresponding polar coordinates (*ρ*, *θ*) description of a solution point **P**. In panel **b**, the amplitude of the field at **P** as it travels the *θ* values along the ellipse for increasing values of *r* defined by the arrow.

To understand the transient imaging model, described mathematically in the Methods section, we start with a single transmitter-receiver pair located, respectively, at **Tx** and **Rx** and sharing the time origin. This means that the receiver starts the acquisition at the same time transmission starts and stops when transmission ends. It thus follows in Fig. 1 that a pulse leaving **Tx**, and scattering at a point **P** before arriving at **Rx** at a time *t*, travels a distance *r* = *ct*. The position of **P** can be anywhere on an ellipse (2D) or ellipsoid (3D). However, the received signal amplitude is proportional to 1/*r*_1_*r*_2_ and thus depends on the position of **P** on the ellipse. The received signal amplitude increases when **P** approaches the axis near **Tx** and **Rx** and decreases otherwise. This difference in amplitude decreases as *t* increases because the ellipse approaches the shape of a circle and thus *r*_1_ ≈ *r*_2_. A signal captured by the receiver at a time *t* can also come from higher-order scattering that happens within the ellipse. For instance, any sequence of scattering points that create a path from **Tx** to **Rx** via a set of *n* connecting vectors {**r**_**1**_, **r**_**2**_, …, **r**_**n**_} will have a corresponding signal amplitude proportional to *W* = 1/*r*_1_*r*_2_…*r*_*n*_ if *r*_1_ + *r*_2_ + ⋯ + *r*_*n*_ = *ct*.

**Fig. 2:**
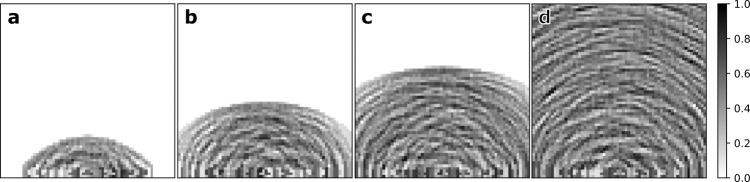
Time-of-flight sampling mask. Panels **a**, **b**, **c**, and **d** show the normalized spatiotemporal mask at different times (*t*_*a*_ < *t*_*b*_ < *t*_*c*_ < *t*_*d*_). This mask is generated by 12 transmitters arranged in a line with the receiver in the center. Notice the several elliptical contributions adding to unique spatial patterns at each time step.

We interpret *W* as the likelihood of finding the scattering point based on the recorded amplitude and time of the pulse capture. As shown in Fig. 1, for a single (**Tx**, **Rx**) pair, there is an ambiguity in the position of this scattering point. This ambiguity can be reduced by adding more transmitters at different positions while keeping a single receiver. Furthermore, each transmitter can also send a sequence of pulses while maintaining the receiver in continuous capture mode. By using different PRBS codes for each transmitter pulse train, this configuration creates a spatiotemporal mask that can probe the whole scene continuously in time (Fig. 2).

The exact number of transmitters and the number of pulses needed to evaluate each voxel in the image depend the on the scene size, number of objects, etc. However, natural scenes are sparse in their primal domain and thus an estimate of the number of pulses/transmitters can be inferred by simple information theory arguments. Sparsity here relates to the presence or absence of free space in the primal scene domain. In this context, we define a scene ${\mathscr{S}}$ as a Cartesian grid comprising *N* voxels where *K* are non-empty. Without any prior knowledge of the scene, we say that the probability of finding a non-empty voxel is *ρ* = *K*/*N*. We consider the scene sparse if *ρ* ≪ 1. Our conjecture is that the number of pulses and transmitters should relate to the Shannon entropy of the scene^11^ *I* = *M*/*N* = −*ρ*log_2_(*ρ*) − (1 − *ρ*)log_2_(1 − *ρ*). *M* is the number of different patterns generated by the spatiotemporal mask and is a quantity related to the number of pulses and transmitters. The sparsity condition thus leads to:

1 $$M\simeq K{{\rm{log}}}_{2}\left(\frac{2N}{K}\right).$$

Because the value of *K* is not known a priori, we calculate *M* by finding the maximum number of patterns a set of transmitters can generate. By allowing each transmitter to emit PRBS pulse codes, for *N*_*T*_ transmitters there are ${2}^{{N}_{T}}$ possible binary combinations that will produce a spatiotemporal mask. With 12 omin-directional antennas, we therefore had access to *M* = 4096 patterns. We also chose to arrange the antenna array in a linear fashion to allow a more convenient access to the PRBS generators. Different number of antennas and their arrangement have not been tested and could produce more optimized results than those described here.

The resulting first-order spatiotemporal mask is illustrated in Fig. 2 where we observe the elliptical contributions from all transmitters aggregated to produce the mask which evolves at approximately half the speed of light. The factor of 1/2 is achieved when the ratio of between *ct* and the ∣**Tx - Rx**∣ distance is large. The discretization of the evolution is dictated by the sampling rate of the receiver.

### Implementation of the imaging model

**Fig. 3:**
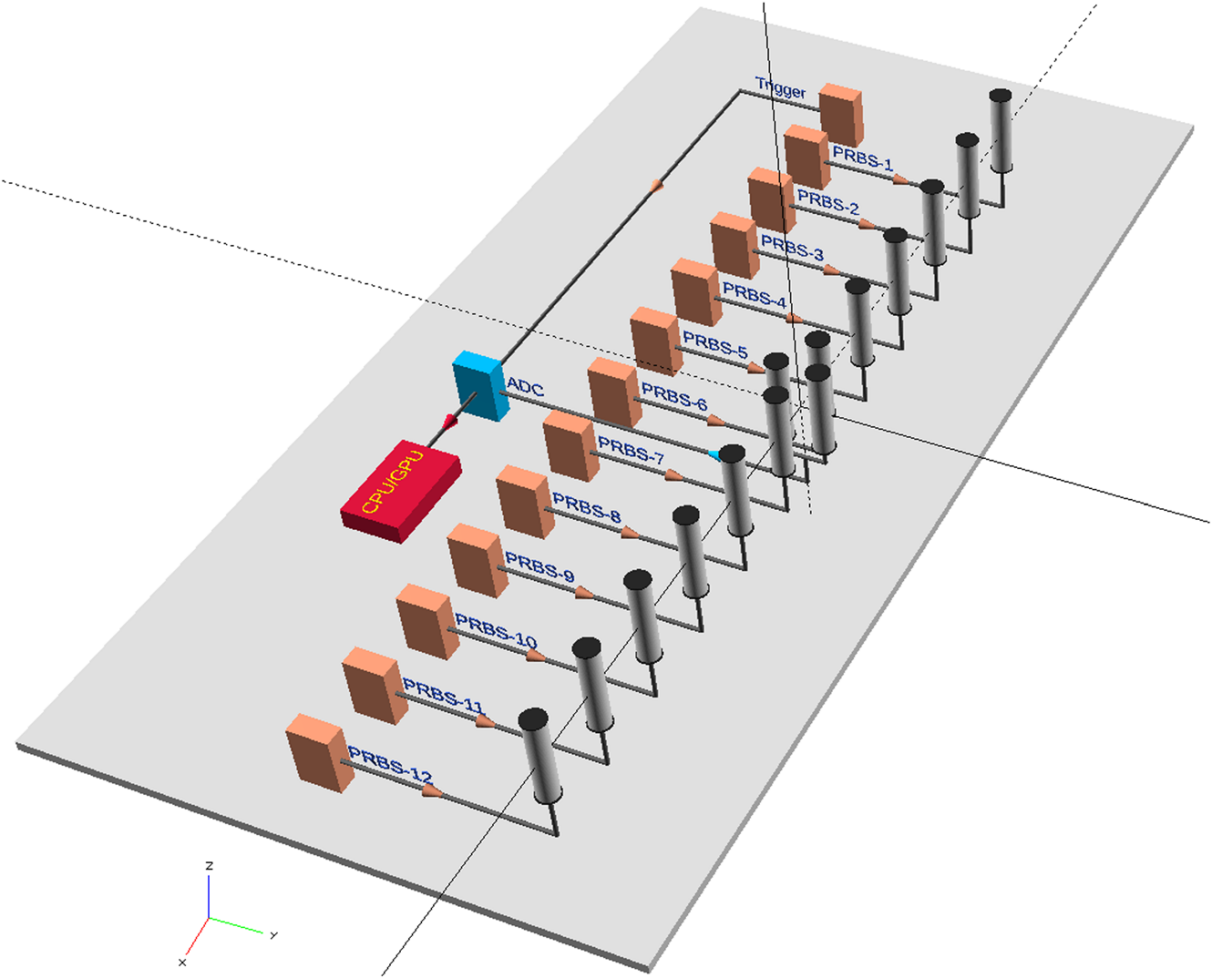
Experimental setup. Schematic showing the pseudo-random binary sequence (PRBS) generators that feed the antenna array after an amplification stage (not shown). The transmitter array comprises 12 omni-directional antennas each receiving a unique PRBS code. The generators are synchronized by the trigger source also used to time align the analog-to-digital converter (ADC). The differential receiver comprises two omini-directional antennas connected by a 180-degree transformer and amplification stage (not shown). The ADC digitizes the signal from the amplified transformer output and stores it in a local buffer. The computer (CPU) queries and transfers the digitized capture from the ADC buffer and uploads it to a graphics processing unit (GPU) that performs the image reconstruction.

Figure 3 shows a schematic diagram of the layout used for the simulation and experiment. We placed 12 omni-directional transmitter antennas along the *x*-axis of a 2D Cartesian coordinate system spanning 5 meters in each direction. The separation between two contiguous antennas was 30.48 cm starting from −1.68 m to +1.68 m. The position of the receiver was at the origin of the coordinate system. In the experiment, the receiver was a pair of omni-directional antennas placed along the y-axis in symmetric positions with respect to the *x*-axis and separated by 15 cm. The output of these antennas fed the input ports of a 180-degree transformer that subtracted their signal. An analog-to-digital converter (ADC) captured the receiver signal in the experiment at a sampling rate *B* defined below. The ADC trigger signal came from the one of the channels of the PRBS generator assigned solely for that purpose. Phase-matched coaxial delay lines ensured the spatiotemporal mask and the signal captures shared the same time origin by making the trigger and captured signals arrive at the same time at the ADC. The pulses in the simulation were bipolar (±1) and single-ended in the experiment. Their capture sampling rate and the emission bit rate *B* was 3 GHz in the simulation and 1.5 GHz in the experiment with sampling time defined as *t*_*s*_ = 1/*B*. This corresponded to a spatial resolution of *δr* = *c*/2*B* of 10 cm for the experiment and 5 cm for the simulation. The number of pixels in each image was the closest power of 2 greater than the spatial resolution: 64 × 64 pixels for the experiment and 128 × 128 pixels for the simulation with a corresponding spatial resolution step (*δr*_∘_) of 7.81 cm and 3.91 cm, respectively. The 2D scene simulations used a finite-difference time-domain package^12,13^. The experimental scene was built inside an electromagnetic anechoic chamber 5 m wide × 5 m tall × 10 m long. The simulated pulses had a 56-point time profile comprising a rising sinusoidal quarter wave edge starting at zero amplitude (12 points), a flat section at amplitude 1 (32 points) and a sinusoidal quarter wave edge starting at amplitude 1 (12 points) and ending one point from amplitude zero. A convolution between this pulse envelope and the PRBS codes for each antenna produced the excitation for each transmitter in the simulation. The simulation time resolution was $\Delta t=\Delta r/c\sqrt{2}$ where the spatial resolution was Δ*r* ≈ 2.52 mm. At a given time, the bits from each antenna formed a 12-bit pattern. The sequence starts with all bits set to one and ends with all bits set to zero. Each intermediate bit pattern is then followed by its complement until all ${2}^{{N}_{T}}$ patterns are used. This choice is an attempt to minimize spectral power below the cutoff frequency of the omni-directional antennas (nominal roll-off 3 dB point at 200 MHz) in the experiment.

The received capture, *y*, contained 4096 points in the experiment and 4096 × 56 in the simulation. A reference capture with an empty scene was subtracted from each capture to remove background signals from the adjacent transmitters. In the simulation we performed a sequential sum in blocks of 56 points to obtain the 4096 samples. The first-order image *x*_1_ was the dot product of the capture *y* and the reconstruction matrix A_1_:

2 $${x}_{1}={{\rm{A}}}_{1}\cdot y.$$

The calculation of the first-order mask A_1_ happens in two steps. First we generate the sampling matrix S_1_ by adding the values of *W* for each transmitter across the *N* voxels in the scene for all *M* sampling and code times. In the next step, we normalize the time series for each voxel by its Euclidean norm ∥ ⋅ ∥_2_. The calculation of A_1_ and A_2_ is described in pseudo-code form in the Methods section.

The calculation of the second-order sampling matrix A_2_ involves an extra loop accounting for the scene voxels weighted by the first-order image mask where the test condition is based on *r*_1_ + *r*_2_ + *r*_3_. The matrix is then normalized in the same fashion as A_1_. The reconstruction of the second-order image requires removing the first-order response from the received signal *y*. This is done by normalizing the first-order image ${x}_{1}^{n}={x}_{1}/\max | {x}_{1}|$ and using the transpose of the first-order reconstruction matrix ${{\rm{A}}}_{1}^{{\rm{T}}}$ to reach:

3 $${y}_{1}=y-{{\rm{A}}}_{1}^{{\rm{T}}}\cdot {x}_{1}^{n}.$$

The second-order image thus follows from Eq. (3) as:

4 $${x}_{2}={{\rm{A}}}_{2}\cdot {y}_{1}.$$

### Simulations

**Fig. 4:**
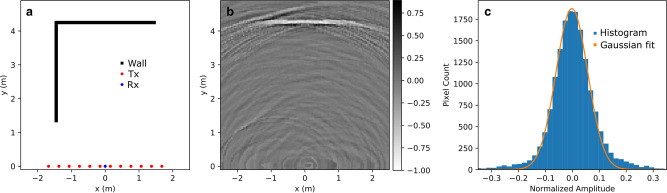
Numerical evaluation of image reconstruction model. The simulated scene in panel **a** shows the two walls (black), the position of the transmitters (red) and the receiver (blue), and the first-order reconstructed image using Eq. (2) with its corresponding gray scale bar in panel **b**. In panel **c**, the histogram of the normalized image pixels in blue is fitted by a Gaussian in orange.

**Fig. 5:**
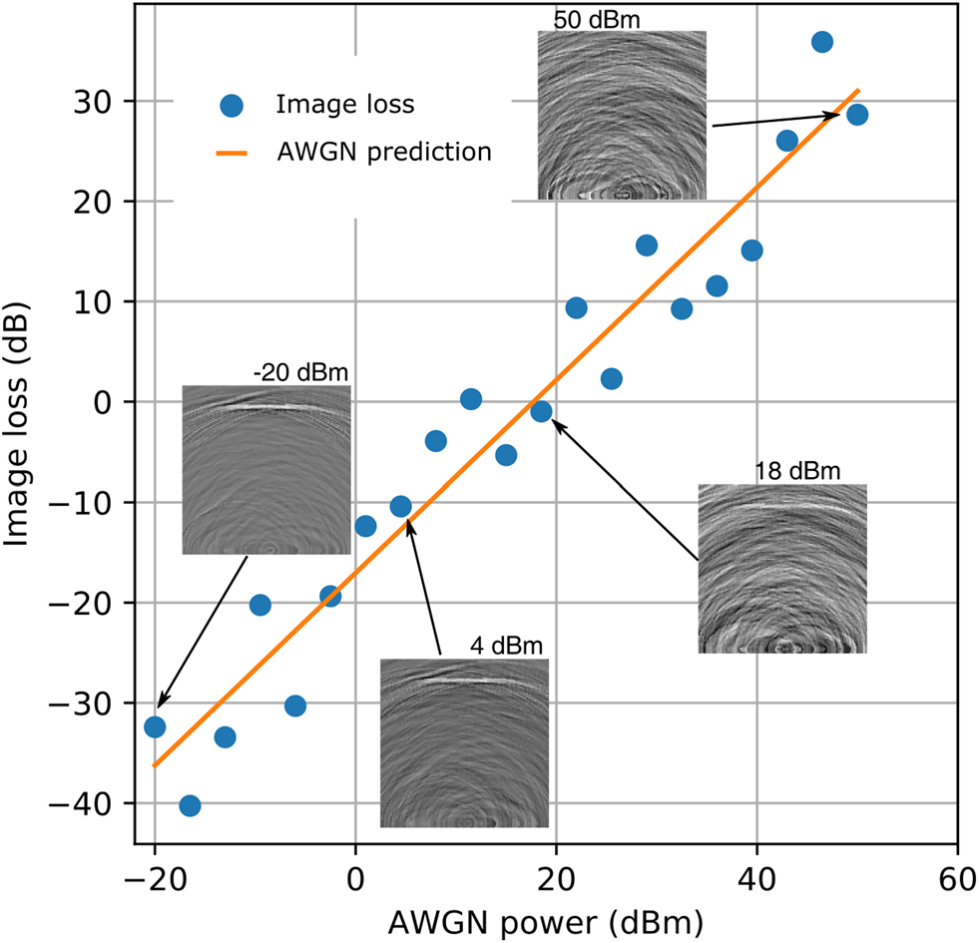
Noise analysis of image reconstruction model. Image loss (blue dots) as a function of additive white Gaussian noise (AWGN) and the AWGN prediction (orange line). Insets show the recovered images at different noise levels for comparison.

We simulated a simple scene comprising two orthogonal walls connected by their ends (Fig. 4a). The walls were 0.1 m in thickness with relative permitivity of *ϵ*_*r*_ = 1.5. The total emission-capture time was 1.37 μs. The first-order reconstructed image contains the horizontal wall but not the vertical wall except for its bottom corner (Fig. 4b). This is because the first-order reconstruction matrix does not contain any second-order signals to correlate with the vertical wall. A histogram of the reconstructed image shows that the voxels are normally distributed at low amplitudes. However, the Gaussian fit does not describe the histogram tails. The Gaussian part of the histogram corresponds to the background of the image, whereas the non-Gaussian tail relates to the horizontal wall and the lower end of the vertical wall. The reconstructed image strength, defined here as −20log_10_(*σ*) is 18.9 dB, where *σ* = 0.11 is the standard deviation of the normalized first-order image voxel values. To evaluate the image strength metric above, we performed an additive white Gaussian noise (AWGN) test. AWGN is a good approximation for the types of noise encountered in most RF hardware in the absence of a detailed specification of the electromagnetic environment at such frequencies^14^. The received signal *y* was normalized to produce 0 dBm of power over the simulation time of 1.37 μs and deviations from the noiseless image were computed for various levels of AWGN. These image loss deviations were computed using the relation 20log_10_∣*I*_*AWGN*_ − *I*_*noiseless*_∣ and measured in dB. *I*_*AWGN*_ and *I*_*noiseless*_ are the reconstructed images with and without AWGN, respectively. Figure 5 shows that the image loss increases linearly with the added noise and that the 0 dB image loss happens around 18 dBm of AWGN.

In addition, the number of non-zero voxels of the threshold mask at 2*σ* is *K* = 722 pixels or 4.4% of the number of pixels in the image. This result corresponds to a value for *M* in Eq. (1) of 3974, which is close to the actual 4096 choice.

**Fig. 6:**
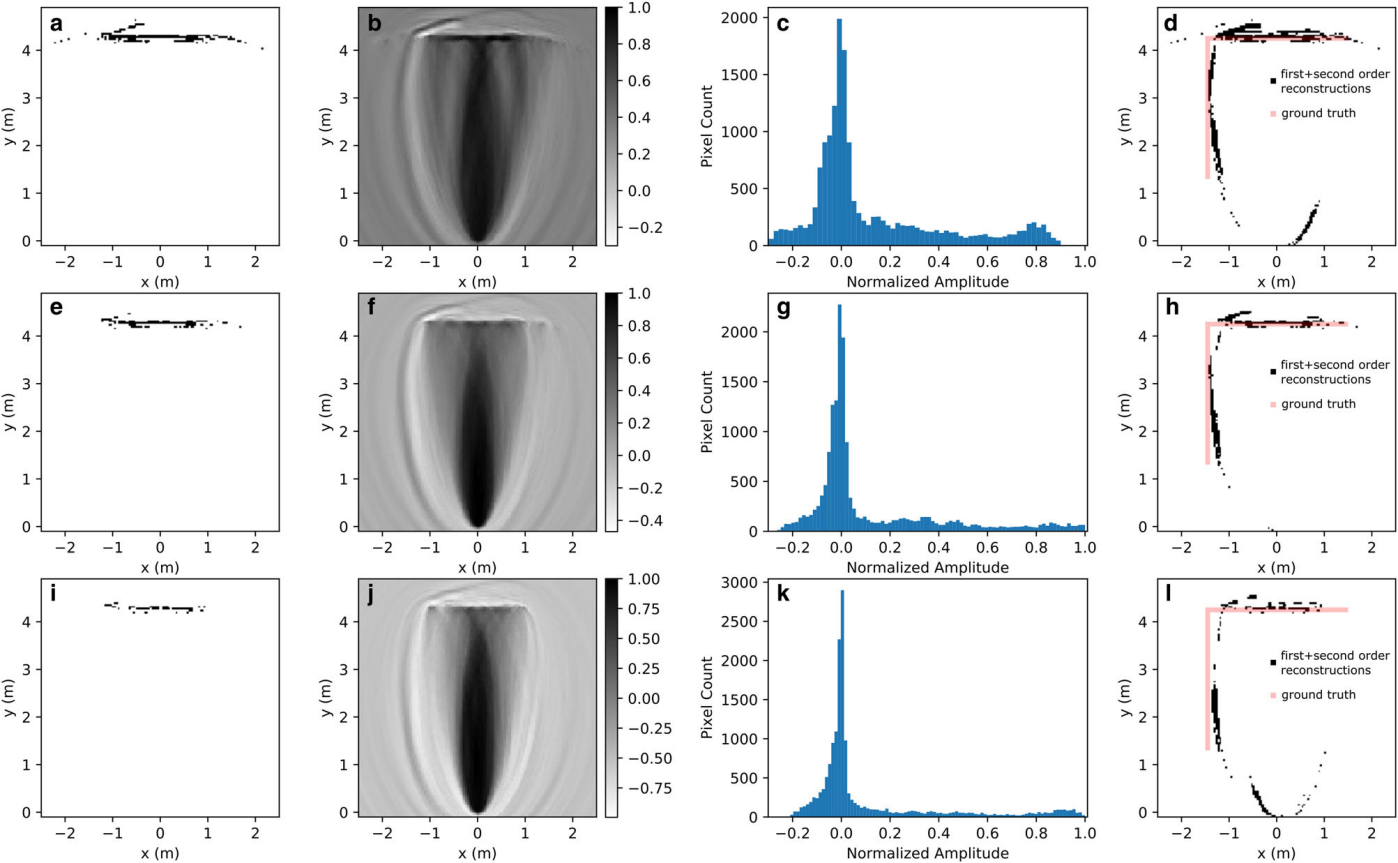
Second-order reconstruction of the scene in Fig. 4a. Panel sets (**a**, **b**, **c**, **d**), (**e**, **f**, **g**, **h**), and (**i**, **j**, **k**, **l**) correspond to threshold filter levels of 4*σ*, 5*σ*, and 6*σ* applied to the normalized image in Fig. 4b, respectively. Panels **a**, **e**, **i** correspond to the filtered first-order image, the second-order result including gray scale bars (**b**, **f**, **j**) and corresponding intensity distribution (**c**, **g**, **k**), and the combined first- and second-order masks (**d**, **h**, **l**) in black with the ground truth scene in red.

To obtain the remainder of the vertical wall, we used the normalized first-order image in Fig. 4b as an input for the calculation of the second-order reconstruction matrix as described above (Fig. 6). We chose three different threshold levels (4*σ*, 5*σ*, and 6*σ*) for the input first-order image mask (panels **a**, **e**, and **i** in Fig. 6). At 4*σ*, the mask contains most of the horizontal wall and some background. At 5*σ*, the mask contains primarily the horizontal wall. Finally, at 6*σ* the mask fails to show the horizontal wall in full. The histograms (Fig. 6, panels **b**, **f**, and **k**) of the normalized second-order images (Fig. 6 panels **c**, **g**, and **k**) show a pronounced peak near zero amplitude followed by nearly flat tails. A simple threshold applied to the those images produce the aggregate image in panels **d**, **h**, and **l** of Fig. 6. The thresholds were set at −0.256, −0.212, and −0.170 for panels **c**, **g**, and **k**, respectively, with only the pixels below the threshold counted. The combined reconstructed images in Fig. 6 panels **d**, **h**, and **l**, show that there is enough specular reflection paths from the transmitters to the receiver that involved two reflections at the horizontal and vertical walls.

### Experiment

**Fig. 7:**
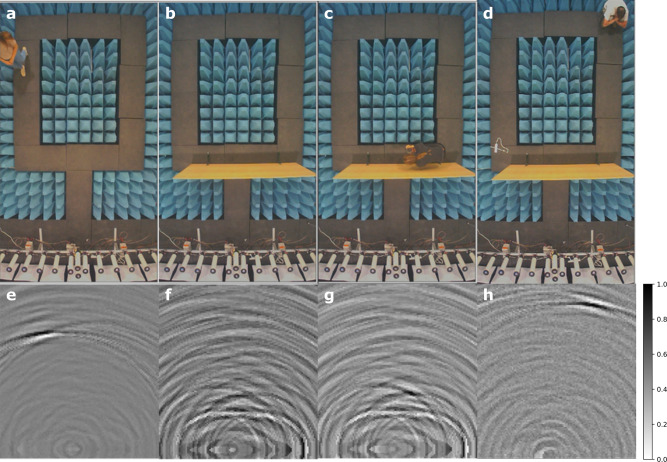
Experimental demonstration. Ground truth images from a camera mounted 5 m above the scene with a person (**a**), a wall (**b**), a person behind a wall and no wall background subtraction (**c**), and a person behind a wall with background subtraction (**d**). The corresponding first-order reconstructions and gray scale bars for panels **a**, **b**, **c**, and **d** set are shown, respectively, in panels **e**, **f**, **g**, and **h**.

As presented in the Supplementary Video, we also performed first-order reconstruction experiments in four different configurations shown as snapshots in Fig. 7. Ground-truth images generated by a camera system installed on top of the anechoic chamber provided direct feedback on the performance of the system under dynamic conditions. In the first configuration (panel **a**) a person walked in front of the antenna array. The performance of the system (panel **e**) is similar to the simulations despite being operated at lower sampling rate (1.5 GHz versus 3 GHz in the simulation). In the second configuration (panel **b**), we placed a particle-board wall (1.22 m tall × 2.44 m wide × 12.7 mm thick) 1 m from the array. We see the image (panel **f**) shows contributions coming from an empty area along the antenna array axis as described in Fig. 1. We then had a person walk behind the wall (panel **c**) in the third configuration. In the real-time screen view of the image (panel **g**), it is possible to see small fluctuations correlate visually with the ground-truth image. However, because the reflection from the wall overwhelms the signal from objects behind it, their signature is difficult to see in the same scale. We thus collected a reference capture from the wall and subtracted it from captures taken with a person in the fourth configuration (panel **d**). The result (panel **h**) shows that the dynamic range of the system is high enough to resolve objects behind the wall.

In the experiment, the capture duration for 4096 samples was 2.73 μs. Due to the on-board buffer memory capability of the ADC, image captures can be recorded at a rate of 1/2.73 μs or 366 kHz for a total duration of 1.3 s. Single-capture data transfers to the computer lasted an average of 0.5 ms per image. The image reconstruction algorithm employed a graphics processing unit that performed *M* × *N* = 4096 × 4096 = 16.8 million operations in 0.9 ms. These performance metrics allow display refresh rates of 700 frames per second or more than 20 times typical video frame rates. The first-order matrix calculation lasted 50 s. If memory of the order *N* × *M* is available, this matrix does not need to be re-calculated unless changes in the position of the antenna array or receiver antenna are made. The second order reconstruction matrix calculations was performed in 9 h on the same graphics processing unit. Because it requires the image from the first-order calculation it poses an optimization challenge. However, if memory of the order *M* × *N*^2^ is available, all second-order contributions can be calculated in no more than 3.6 s using the above GPU hardware.

## Discussion

We developed an RF transient imaging model that uses continuous emission and capture to obtain images with a few microseconds of capture time. Simulations and experiments demonstrated its dynamic range, spatial and time resolution to produce through-wall images in real-time. The dynamic range of the system is sufficient to resolve small changes in position and possibly shape variations at sub-pixel resolutions. The reconstruction matrix operates on each voxel and can be calculated to image patches in a wider range in order to save memory. The above results show that this system can be used for a variety of applications involving real-time analysis. These include tracking explosions^15^ and space debris dynamics^16^ where objects travel at speeds of the order of 10 km/s.

The transient image model is a full 3D model that can be used to produced 2D images without any loss of generality. In the case of the experimental images, each voxel represented a projection of all scatterings happening in the *z*-axis. To remove *z*-axis ambiguities, a 2D emitter array must be employed.

This approach also demonstrated second-order images under specular conditions. It is important to mention, however, that the specular conditions are very stringent and second-order reflections may not be sufficient to recover all the details in a scene. In fact, without structuring the first-order and characterizing higher-order responses as done by Liu^8^, the second-order reconstruction presents some challenges. However, if the specular conditions are relaxed and diffuse scattering is present as is the case in some optical systems, second-order imaging may restore more of the overall components of a scene.

## Methods

### Derivation of the transient image model

To generate the reconstruction matrix we used a simple light transport model based on the propagation and scattering of Dirac delta functions. We start with the wave equation:

5 $$\frac{1}{{c}^{2}}\frac{{\partial }^{2}E}{\partial {t}^{2}}-{\nabla }^{2}E=F(t,{\bf{r}},{t}_{j},{{\bf{r}}}_{{\bf{j}}}).$$

The solution for a point source *F* = *δ*(*t* − *t*_*j*_, **r** − **r**_**j**_) localized in space and time is the Green’s function^17^:

6 $$G(t,{\bf{r}},{t}_{j},{{\bf{r}}}_{{\bf{j}}})=\frac{1}{4\pi | {\bf{r}}-{r}_{{\bf{j}}}| }\delta \Big[{t}_{j}-\Big(t-\frac{| {\bf{r}}-{{\bf{r}}}_{{\bf{j}}}| }{c}\Big)\Big].$$

In Eq. (6), *δ* is the Dirac delta function. The above expression can be extended to an array of *N*_*T*_ point sources emitting pulses at random times according to a pseudo-random binary sequence *C*_*j*_(*t*_*j*_):

7 $${E}^{(\circ )}(t,{\bf{r}})=\mathop{\sum }\limits_{j=1}^{{N}_{T}}{C}_{j}({t}_{j})G(t,{\bf{r}},{t}_{j},{{\bf{r}}}_{{\bf{j}}}).$$

In terms of the transient rendering^6^, *E*^(∘)^ is the locally emitted flux. It follows that the first-order response of the scene *E*^(1)^ upon illumination by *E*^(∘)^ is given by the geometry and visibility term *x*, and a scattering term represented by the Green’s function (Eq. (6)):

8 $${E}^{(1)}(t,{\bf{r}})=\int {E}^{(\circ )}(t^{\prime} ,{\bf{r}}^{\prime} )G(t,{\bf{r}},t^{\prime} ,{\bf{r}}^{\prime} ){x}_{1}({\bf{r}}^{\prime} )dt^{\prime} {\bf{d}}r^{\prime} .$$

The Dirac delta functions in Eq. (8) will make the integral in $dt^{\prime}$ over the sampling time *t*_*s*_ vanish except when the path connecting a transmitter to a point in the scene (**r**_**1**_) and from that point to the receiver (**r**_**2**_) satisfies the condition *r*_1_ + *r*_2_ = *ct*. The integral in $d{\bf{r}}^{\prime}$, when evaluated over the spatial resolution step *δr*_∘_ across the scene, leads to the the dot product of the S matrix and the discretized version of *x*. Each entry of S is a sum of the non-zero likelihood factors 1/*r*_1_*r*_2_. Finally, the value of the received signal is evaluated at the origin of the coordinate system *y* = *E*^1^(*t*, **r** = 0) giving the first-order transient rendering equation:

9 $$y={{\rm{A}}}_{1}\cdot {x}_{1}.$$

Notice that *y* already has the background contribution from *E*^(∘)^ subtracted as in the case of the simulation and experimental data. The resulting value of *x*_1_ is the first-order term in an expansion. It does not contain components due to multiple scatterings. To obtain higher order corrections to *x*, we calculate *E*^(1)^ using the first-order solution to solve for *x*_2_ in the second-order integral:

10 $${E}^{(2)}(t,{\bf{r}})=\int {E}^{(1)}(t^{\prime} ,{\bf{r}}^{\prime} )G(t,{\bf{r}},t^{\prime} ,{\bf{r}}^{\prime} ){x}_{2}({\bf{r}}^{\prime} )dt^{\prime} d{\bf{r}}^{\prime} .$$

The expansion to higher orders follows by induction:

11 $${E}^{(n)}(t,{\bf{r}})=\int {E}^{(n-1)}(t^{\prime} ,{\bf{r}}^{\prime} )G(t,{\bf{r}},t^{\prime} ,{\bf{r}}^{\prime} ){x}_{n}({\bf{r}}^{\prime} )dt^{\prime} d{\bf{r}}^{\prime} .$$

### Reconstruction algorithm

The algorithm below includes first- and second-order effects. The *Image*[*pixel*] variable is the image calculated in the first-order pass of the algorithm where line 27 must read “Add 0 to *scene*”. For the second-order calculation, line 19 must read “Add 0 to *scene*”. The algorithm was scripted in Python with the GPU components written in C^18^ for an Nvidia Titan V GPU.

#### Algorithm 1

Reconstruction matrix calculation in 2D

1: Initialize coordinates for the transmitters and receiver

2: Initialize sampling time array with *M* elements

3: Initialize the codes matrix for all transmitters ${C}_{{N}_{T}\!{\times}\!M}$

4: Initialize *scene* with *N* elements

5: Initialize *S*_*M*×*N*_

6: Initialize A_*N*×*M*_

7: **for** every $t^{\prime}$ in the sampling time array **do**

8: Set all elements of *scene* to zero

9: **for** every transmitter coordinate **r**_**Tx**_ **do**

10: **for** every time *t*_*Tx*_ in the transmitter code time array **do**

11: **if** $$t=t^{\prime} -{t}_{Tx}\ge 0$$ **then**

12: Set *r*_*min*_ = *c*(*t* − *t*_*s*_)

13: Set *r*_*max*_ = *ct*

14: **for** every pixel position in *scene* **do**

15: Calculate the distance from pixel to transmitter *r*_1_

16: Calculate the distance from pixel to receiver *r*_2_

17: Calculate *r* = *r*_1_ + *r*_2_

18: **if** *r*_*min*_ ≤ *r* < *r*_*max*_ **then**

19: Add ${C}_{{T}_{x},t}/{r}_{1}{r}_{2}$ to *scene*

20: **else**

21: **for** every pixel position in *scene* **do**

22: Calculate the distance from first pixel to transmitter *r*_1_

23: Calculate the distance from first to second pixel *r*_2_

24: Calculate the distance from second pixel to receiver *r*_3_

25: Calculate *r* = *r*_1_ + *r*_2_ + *r*_3_

26: **if** *r*_*min*_ ≤ *r* < *r*_*max*_ **then**

27: Add $Image[pixel]\times {C}_{{T}_{x},t}/{r}_{1}{r}_{2}{r}_{3}$ to *scene*

28: **end** **if**

29: **end** **for**

30: **end** **if**

31: **end** **for**

32: **end** **if**

33: **end** **for**

34: **end** **for**

35:**end** **for**

36: **for** Every pixel *n* in *scene* **do**

37: Calculate A_n,M_ = S_M,n_/$$\sqrt{{\sum }_{{\rm{n}}}{{\rm{S}}}_{{\rm{M,n}}}^{2}}$$

38: **end** **for**

## Supplementary information

Description of Additional Supplementary Files

Supplementary Movie 1

## Data Availability

Data used in the preparation of this manuscript is available in the text, supplemental material or upon reasonable request to the first author.

## Code availability

The code needed to reproduce the results of this manuscript is presented in the manuscript as pseudo-code.

## Disclaimers

Certain commercial equipment, instruments, or materials are identified in this paper in order to specify the experimental procedure adequately. Such identification is not intended to imply recommendation or endorsement by NIST, nor is it intended to imply that the materials or equipment identified are necessarily the best available for the purpose. This is an official contribution of the National Institute of Standards and Technology; not subject to copyright in the United States.

## References

[1] Bhandari A, Raskar R (2016). Signal processing for time-of-flight imaging sensors. IEEE Signal Process. Mag..

[2] Faccio D, Velten A (2018). A trillion frames per second: the techniques and applications of light-in-flight photography. Rep. Prog. Phys..

[3] Ghasr MT, Horst MJ, Dvorsky MR, Zoughi R (2017). Wideband microwave camera for ream-time 3-d imaging. IEEE Trans. Antennas Propag..

[4] Charvat G, Temme A, Feigin M, Raskar R (2015). Time-of-flight microwave camera. Sci. Rep..

[5] Velten A (2012). Recovering three-dimensional shape around a corner using ultrafast time-of-flight imaging. Nat. Commun..

[6] Smith, A., Skorupski, J. & Davis, J. Transient rendering. *Technical Report UCSC-SOE-08-26* https://www.soe.ucsc.edu/research/technical-reports/UCSC-SOE-08-26 (2008).

[7] Jarabo A, Masia B, Marco J, Gutierrez D (2017). Recent advances in transient imaging: a computational graphics and vision perspective. Vis. Informatics.

[8] Liu X (2019). Non-line-of-sight imaging using phasor-field virtual wave optics. Nature.

[9] Adib F, Hsu C, Mao H, Katabi D, Durand F (2002). Capturing the human figure through a wall. ACM Trans. Graph..

[10] Seitz SM, Matsushita Y, Kutulakos KN (2005). A theory of inverse light transport. Tenth IEEE International Conference on Computer Vision (ICCV’05) Volume 1.

[11] Shannon C (1948). A mathematical theory of communication. Bell System Technical J..

[12] Warren C, Giannopoulos A, Giannakis I (2016). Open source software to simulate electromagnetic wave propagation for ground penetrating radar. Comput. Phys. Commun..

[13] Warren C (2018). A cuda-based gpu engine for gprmax: open source fdtd electromagnetic simulation software. Comput. Phys. Commun..

[14] Goldsmith, A. *Wireless Communications* (Cambridge Univ. Press, 2005).

[15] Pooley J, Price E, Ferguson J, Ibsen M (2019). Detonation velocity measurements with uniform fibre bragg gratings. Optics Express.

[16] Mehrholz D (2002). Detecting, tracking and imaging space debris. ESA Bulletin.

[17] Felsen, L. & Marcuvitz, N. *Radiation and Scattering of Waves* (Prentice-Hall, 1972).

[18] Klöckner A (2012). PyCUDA and PyOpenCL: a scripting-based approach to GPU run-time code generation. Parallel Comput..

