# Supplementary material for: Continuous-capture microwave imaging
Source: Nat Commun. 2021 Jun 25;12:3981. doi: 10.1038/s41467-021-24219-0 (PMC8233389; doi:10.1038/s41467-021-24219-0)
Supplement: Supplementary file 1 — Description of Additional Supplementary Files [file 41467_2021_24219_MOESM1_ESM.docx]

Description of Additional Supplementary Files

Title: Supplementary Movie 1

Description: Experimental demonstration video of the imaging system showing the original scene and the corresponding reconstructed image.
